# Validation of a Broadband Tissue-Equivalent Liquid for SAR Measurement and Monitoring of Its Dielectric Properties for Use in a Sealed Phantom

**DOI:** 10.3390/s20102956

**Published:** 2020-05-23

**Authors:** Andrew P. Gregory, Kristell Quéléver, Djamel Allal, Ourouk Jawad

**Affiliations:** 1 National Physical Laboratory (NPL), Hampton Road, Teddington TW11 0LW, UK; 2 ART-Fi SA, 2 Rue Jacques Monod, 91400 Orsay, France; kristell.quelever@art-fi.eu (K.Q.); ourouk.jawad@art-fi.eu (O.J.); 3 Laboratoire National de Métrologie et d’Essais (LNE), 29 Avenue Roger Hennequin, 78190 Trappes, France; djamel.allal@lne.fr

**Keywords:** dielectric measurement, process monitoring, open-ended coaxial probe, specific absorption rate (SAR), tissue-equivalent materials

## Abstract

We report on the development of a method for measuring the permittivity and conductivity of fluids inside a sealed tank (or a pipe) by using an embedded coaxial probe. Permittivity and conductivity in the frequency range 600 MHz to 6 GHz are determined from measurements of a complex reflection coefficient by using a vector network analyser (VNA) that is connected to the embedded probe via a coaxial cable. Substitution methods for calibration of an inaccessible probe are studied in this paper. These require the VNA with attached cable to be calibrated prior to connecting the cable to the embedded coaxial probe. Measurement of permittivity and conductivity of fluids inside sealed tanks and pipes is needed for monitoring industrial processes, such as fermentation. The authors’ requirement, however, was to allow monitoring of a tissue-equivalent liquid that is contained inside a sealed tank. This tank is a component of a commercial system for rapid, multiple-band measurement of the specific absorption rate (SAR) of mobile phone handsets. Monitoring of permittivity and conductivity is needed to ensure compliance with international standards for SAR measurement. The paper also presents data for a new broadband (600 MHz to 6 GHz) tissue-equivalent liquid that is based on an oil-in-water emulsion. It is demonstrated that over an extended period of time, the liquid is stable, and an embedded coaxial probe enables its properties to be monitored with the required accuracy.

## 1. Introduction

There are multiple applications for online measurement of the dielectric permittivity of fluids inside pipes and tanks. These include monitoring of fermentation processes in the manufacture of food and beverages [1,2,3], monitoring polymerisation reactions [4], and observation of multiphase samples flowing in pipes, e.g., to allow oil to be discerned from water in the petrochemical industry [5,6,7]. This paper describes a measurement technique that uses a coaxial probe that is embedded in a tank or pipe, and not removable for calibration. The technique enables broadband measurements at microwave frequencies on high-loss materials, including polar liquids such as water. Therefore, it is complementary to resonant methods that enable the measurement of the dielectric loss of low loss materials, such as oils [7,8]. The application of the technique for one particular purpose—for monitoring the stability of a phantom liquid (a high-loss liquid that has properties that are similar to those of head tissue) enclosed in a sealed tank—is studied. Sealed phantoms are used in instrumentation for type-approval testing of emitting devices such as mobile phone handsets to show that the specific absorption rate (SAR) is in within the permitted limits.

The developments described in this paper support new vector methods for rapid measurement of SAR that are the subject of a new international standard [9]. Reference [10] describes a system for vector measurement of SAR that uses a multiplexed array of antennas that are embedded in a polymer tank (0.4 m × 0.3 m × 0.2 m) that is filled by a broadband tissue-equivalent liquid. Vector measurements are used to obtain images of the electric fields. Through this approach, the time to determine the peak SAR (1 or 10 g average) of a handset is reduced by a factor of at least 100 compared to robot-based scanning systems described in earlier standards [11,12,13,14]. The reduction in the time required for testing is particularly significant for modern smartphones, as these have more than 30 transmitting modes (technologies and bands) that in total would require typically five weeks of continuous testing with a robot-based scanning system. This paper does not focus on SAR measurement itself, but describes instrumentation for monitoring the dielectric properties of phantom liquids (and liquids in general) inside sealed tanks. The paper also presents measurements on a phantom liquid to show that (i) it is stable and (ii) it meets the specifications required by international standards over the frequency range from 600 MHz to 6 GHz.

**Figure 1:**
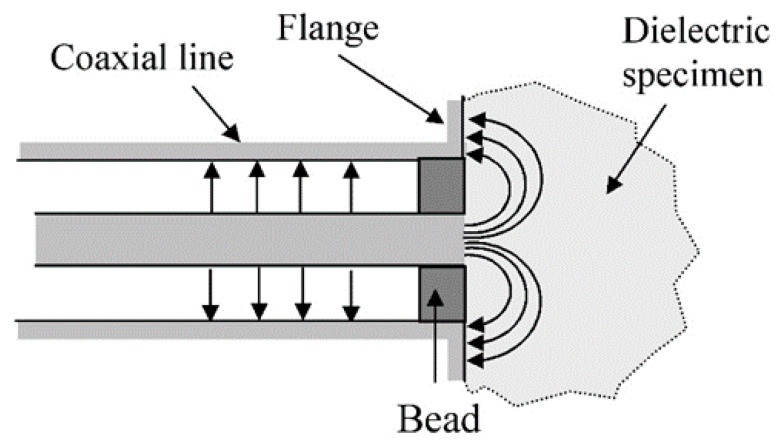
Schematic of coaxial sensor. Complex permittivity is determined from the complex reflection coefficient measured by using a vector network analyser (VNA).

In the field of SAR measurement, it is conventional practice to specify the dielectric properties [15] of tissue-equivalent liquids by the real part ε′ of the relative permittivity (often referred to only by ‘permittivity’) and the total conductivity σ = ωε_o_ε″ (where ε″ is the imaginary part of the relative permittivity, ω is the angular frequency and ε_o_ is the permittivity of free space). Liquids used in SAR compliance testing must comply with target specifications for ε′ and σ given by standards and summarised in a report [16]. Formulations are usable over a range of frequencies as the specifications have ±10% tolerance. Broadband phantoms [17,18] that comply with the specifications over an extended frequency range simplify SAR testing of multiband devices. Open-ended coaxial probes (also known as coaxial sensors) [19,20] have for many years been used for measuring these properties. Figure 1 shows a schematic of a coaxial sensor. They are well-suited for measurements on high-loss materials such as tissue-equivalent liquids at microwave frequencies, are commercially available, and are simple to use. Measurements of ε′ and σ are calculated from the complex reflection coefficient (S11) data that is obtained by using a vector network analyser (VNA). Before a coaxial probe can be used for measurement, a calibration process is required to define a reference plane at the face of the probe and correct for mismatches. The most widely used calibration process [20] is based on short-circuit, air and reference liquid [21] measurements. For a general overview of methods for measuring the permittivity of lossy liquids, see reference [22].

The conventional coaxial-probe method cannot be used to measure ε′ and σ of a liquid in a sealed tank as calibration requires access to the face of the probe. The possibility of sensing through the polymer wall of the tank with a large-sized coaxial probe placed externally (i.e., a multilayer geometry [23]) was considered, but modelling showed that for the type of tank used for SAR measurement (wall thickness 2-mm), the measurement uncertainty would be unacceptably large. The possibility of using a resonance method [24,25,26], based on a device that has a split-ring or similar structure, was considered. While such devices are interesting, they were considered not to be well suited to the present application. Instead, it was decided to adapt the coaxial-probe method to enable calibration by substitution by using a second probe, so access to the face of the probe embedded in the tank is not required. This approach retains the main advantages of the coaxial probe technique: broadband frequency coverage, well-established suitability for measuring liquids with high dielectric loss, use of a calculable geometry with well-tested analytical software based on modal analysis [27], simple construction, traceability of measurements, and the availability of tested procedures for the evaluation of uncertainty [20]. The substitution technique assumes that the properties of the two coaxial probes are constant, and are not changed by contact with samples. As they are simple devices consisting only of metal coaxial conductors and a sealed-in polymer insert (known as the ‘bead’) this is a viable proposition.

Section 2 of this paper describes an open-ended coaxial probe that is designed to be embedded in a tank such as the sealed phantom. The substitution method of calibration is also described in detail. In Section 3.1, measurements of ε′ and σ of a broadband tissue-equivalent liquid that is suitable for the range from 600 MHz to 6 GHz are presented. The liquid is shown to be stable enough to have a long life so that a sealed phantom that requires little maintenance can be made with it. In Section 3.2, the accuracy of measurements of a probe calibrated by the substitution method of calibration studied. It is found that the method enables precise measurement.

## 2. Materials and Methods

The method evaluated in this paper uses two well-matched open-ended coaxial probes, Probe A and Probe B. The methodology developed can be applied when Probe A is freely-accessible, but Probe B, after an initial characterization process, is embedded permanently in a sealed tank or pipe. The actual measurements reported were made in laboratory conditions in which measurements with both probes were made on samples in open beakers. The process of substitution calibration can be described as follows: Probe A is calibrated by the reference liquid method [20] (which will be described below). The VNA test-port cable is then connected to Probe B, which is then used for measurements. While the concept of substitution methods for calibrating open-ended coaxial probes is not new, the technique described in this paper differs from previous work [19] in that it is combined with a reference liquid method of calibration.

If the probes were identical then after the substitution no further action would be required in order to obtain traceable measurements. For the work described in this paper, an additional step (which will be described in Section 2.1) was needed to account for differences between the probes. A variation on the calibration method that uses coaxial standards instead of Probe A is described in Section 2.2 Measurements demonstrating that the substitution method can enable accurate measurements are given in Section 3.

**Figure 2:**
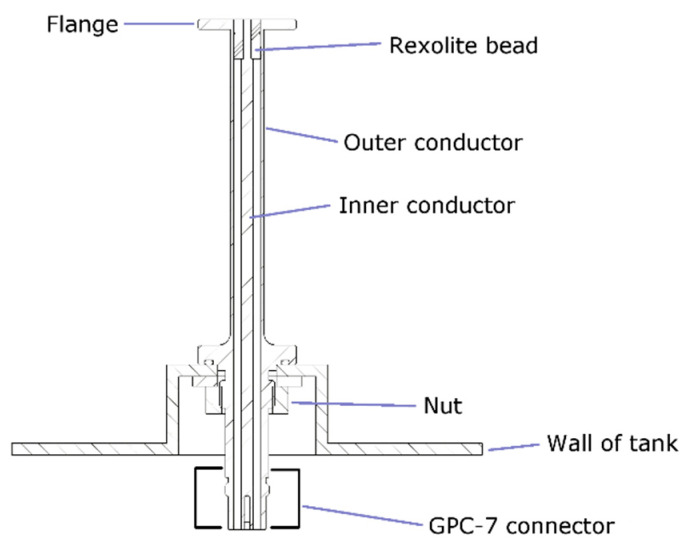
Design of the 7-mm open-ended coaxial probes that are used for monitoring a liquid in a sealed tank. A calibration scheme that takes account of the inaccessibility of the flange is required.

**Figure 3:**
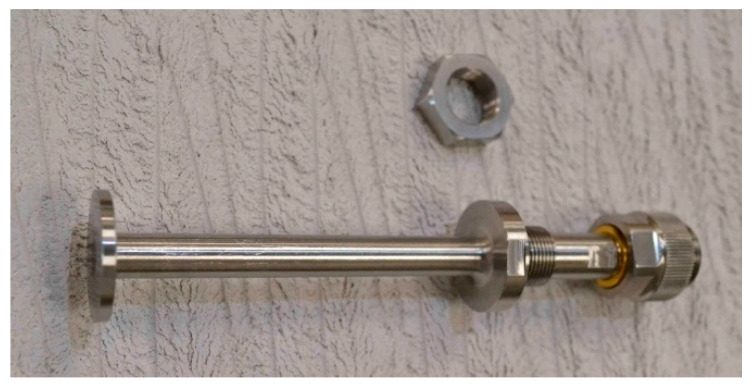
Photograph of one of the 7-mm open-ended coaxial probes. Probe A is shown, but Probe B is of identical design.

The two open-ended coaxial probes, which are nominally identical, were manufactured at the National Physical Laboratory (NPL) according to the design shown in Figure 2. A photograph of one of the probes is shown in Figure 3. They are based on 7-mm coaxial line and are similar to an earlier design [20], but longer and with fittings appropriate for a tank. The flange diameter is 25 mm. A VNA is connected to the probe via a metrology-grade phase-stable cable. A GPC-7 (General Precision 7 mm Connector, also referred to by GPC-7 and PC-7) connector is chosen as this allows very repeatable connections to be made (variation of reflection coefficient magnitude is typically ±0.001 Units). Flange resonances are known to occur for this size of probe if high permittivity (polar) liquids that have loss tangent, ε″/ε′, ⪝ 0.3 are measured [20]. They are not accounted for by the mathematical model used [27] (which is developed for a flange of infinite diameter) and so are a cause of error.

The probes can be calibrated individually by using measurements on a short-circuit, an open-circuit and a reference liquid. Checks on the repeatability of short-circuit measurements were made using calibration procedures described in reference [20]. Two reference-liquids are used for calibration: methanol is used for the upper part of the band (2.5–6 GHz) and ethanol for the lower (<2.5 GHz). The reason for not using methanol throughout the band is that a small (but observable) flange resonance occurs at ~2 GHz for this size of probe. The permittivity ε′ of ethanol is rather low above a few GHz, so best results are obtained by using the two reference liquids in combination. Water cannot be used for calibrating the 7 mm coaxial probes on account of flange resonances. Tables of traceable complex permittivity data for reference liquids as a function of temperature are available [21]. Modal analysis software [27] that allows the complex reflection coefficient to be calculated from the dimensions of the probe and the complex permittivity of the sample was used throughout the calibration and measurement processes.

### 2.1. Equations for the Substitution Calibration

Initial experiments showed that after re-connecting the VNA cable from Probe A to Probe B to perform the substitution the measured |*S*_11_| changed by up to 0.02 units. Measurements on air were used for this test. To improve the accuracy of measurements on a fluid in a sealed tank, coefficients for an additional correction that accounts for differences between the probes can be obtained by the following process:

(1) Probe A is calibrated by using short-circuit, open-circuit and reference liquid standards by the conventional manner. This determines the complex calibration coefficients d, e and f of the bilinear equation:
  (1) $$\Gamma_{A}=\frac{d\Gamma +e}{f\Gamma +1},$$
  in which Γ is the uncorrected complex reflection coefficient (*S*_11_) measured by the VNA, and $\Gamma_{A}$ is the corrected complex reflection coefficient for a reference plane at the face of probe A.
(2) Probe B is attached to the same VNA port and is also calibrated by using short-circuit, open-circuit and reference liquid standards. However, there is an important difference: the data read from the VNA is already corrected by the Probe A calibration. The corrected complex reflection coefficient for a reference plane at the face of Probe B is given by the bilinear equation:
  (2) $$\Gamma_{B}=\frac{a\Gamma_{A}+b}{c\Gamma_{A}+1},$$
  where a, b and c are complex calibration coefficients. These coefficients are retained for use after Probe B has been embedded in the tank. Note that if the probes match perfectly, then a=1 and b=c=0.

To apply the substitution calibration, Probe A is calibrated conventionally by the reference liquid to determine the coefficients d, e and f. Then, using the stored coefficients a, b and c, Equations (1) and (2) are combined to produce a new calibration equation, also of bilinear form, that relates $\Gamma_{B}$ to Γ. The measurement process is eased by the use of software that can convert sets of bilinear coefficients to the form used by the VNA (*E*_D_, *E*_S_ and *E*_R_), and which then transfers them to the VNA via a GPIB interface. Probe B can then be attached to the same VNA port, enabling calibrated measurements on the liquid inside the tank to be obtained.

### 2.2. A Variation on the Substitution Method That Uses GPC-7 Coaxial Standards

**Figure 4:**
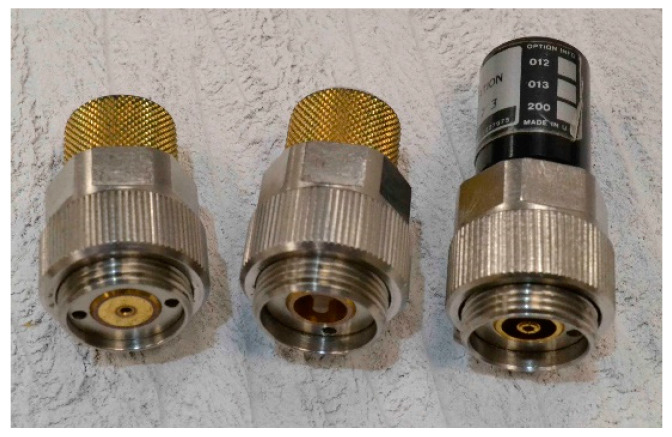
GPC-7 impedance standards (left to right: short-circuit, open-circuit and low-band load).

A variation on the above technique uses coaxial GPC-7 standards (Figure 4) to calibrate at the end of the VNA cable prior to attaching Probe B. In this case, Probe A is not required. An initial reference liquid calibration for Probe B is needed to determine the calibration coefficients a, b and c of Equation (2) before it is embedded in the tank.

## 3. Results

### 3.1. Characterisation of a New Broadband Phantom Liquid

A new human-tissue-equivalent phantom liquid based on oil-in-water emulsion has been developed at the ART-Fi company [17]. This liquid is used to fill a polymer tank to make a sealed phantom that complies with IEC 62209 standards [13,14] over a broad band of frequencies. The formulation includes a biopolymer that is added to the emulsion to produce a viscoelastic fluid with a weak gel-like structure. This fluid exhibits a low yield stress (2.5 Pa) that enables the fluid to flow at very low shear rate. The biopolymer has little effect on the dielectric properties of the liquid, but improves its colloidal stability and minimises air bubbles.

**Figure 5:**
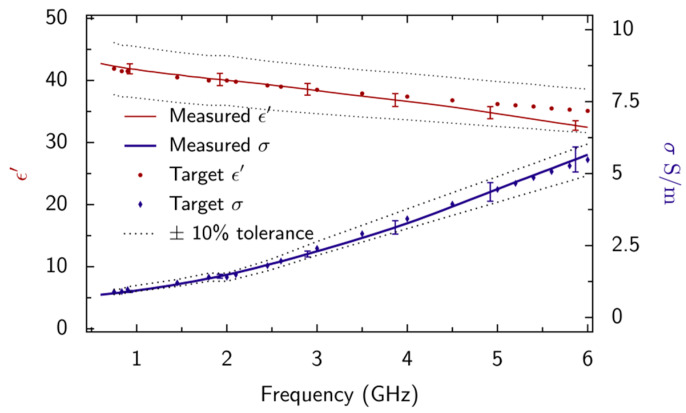
Dielectric properties of the broadband phantom liquid and IEC 62209 target values [13,14] between 600 MHz and 6 GHz, at 22 °C. The observed Type A uncertainty associated with measurements made by using the 85070E 3.5 mm probe (Table 1) is also plotted for a coverage factor of *k* = 2 (equivalent to 95% confidence level).

The dielectric properties, ε′ and *σ*, of the new phantom liquid were measured at a controlled temperature (22 ± 0.5 °C) using an 85070E high-temperature open-ended coaxial probe (3.5 mm aperture) from Agilent Technologies. The manufacturer’s software was used for making these measurements. The probe was calibrated by using an open-circuit measurement, a short-circuit measurement and a measurement on a reference liquid (deionized water). The phantom liquid meets the ±10% tolerance accepted by standards for head and body phantoms at ambient temperature over the broadband frequency range from 600 MHz to 6 GHz (Figure 5). Table 1 provides evaluations of the Type A uncertainty that were evaluated at ART-Fi. The probe manufacturer provides a generic specification [28] of accuracy of 5% for both real and imaginary parts of complex permittivity.

#### 3.1.1. Measurement of Stability over a Period of Time

**Figure 6:**
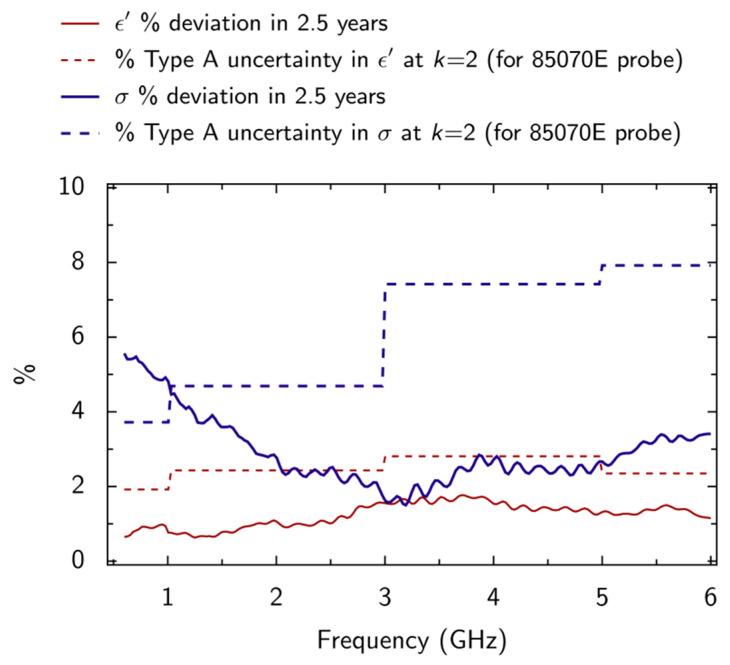
Observations of the effect of ageing (by a 2.5-year interval) on the dielectric properties ε′ and *σ* of the broadband tissue-equivalent liquid. The observed Type A uncertainty associated with measurements made by using the 85070E 3.5 mm probe (Table 1) is also plotted for a coverage factor of *k* = 2 (equivalent to 95% confidence level).

The stability is estimated (1) visually as phase separation occurs over a prolonged period, and (2) by measuring the evolution of dielectric properties over time. If kept in a sealed container, phantom liquid is observed to remain stable for several years. Figure 6 shows how the dielectric properties ε′ and *σ* of a sample of the liquid were observed to change in a 2.5-year interval. These measurements were made by using an 85070E 3.5 mm probe. The observed changes were smaller than the Type A measurement uncertainty (Table 1), except for *σ* values at frequencies between 600 MHz and 1 GHz. However, it can be observed that despite this deviation *σ* remains within the specification given by the standard throughout the measured range, 600 MHz to 6 GHz. No separation of the emulsion was observed to occur during the 2.5-year interval.

#### 3.1.2. Inter-Laboratory Measurement Comparison

**Figure 7:**
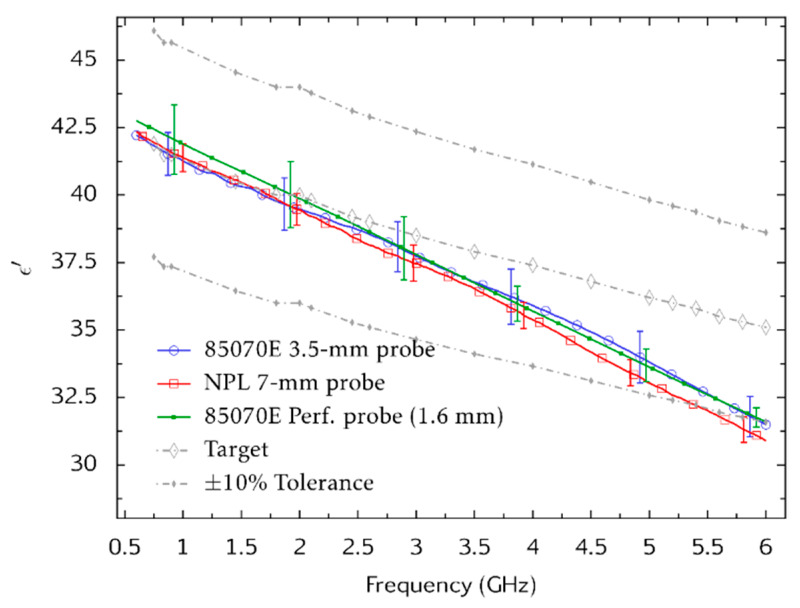

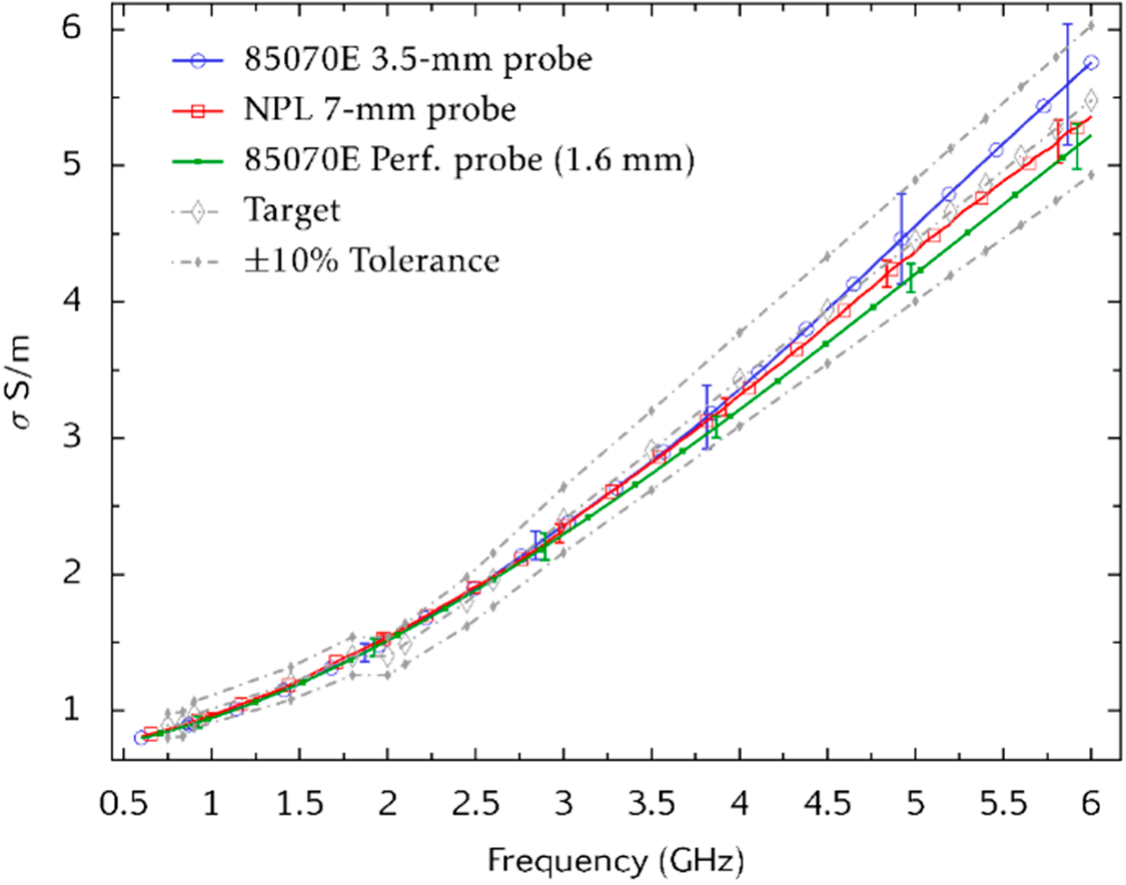
Inter-laboratory comparison of tissue-equivalent liquid dielectric properties. Measurements of the dielectric properties ε′ (**top**) and *σ* (**bottom**) are shown. Uncertainties for the 85070E probes are taken from the manufacturer’s specification. Combined Type A and Type B uncertainty for the National Physical Laboratory (NPL) 7 mm probe was evaluated by Monte-Carlo modelling [20]. The observed Type A uncertainty associated with measurements made by using the 85070E probes are also plotted. All uncertainties are presented at a coverage factor of *k* = 2 (equivalent to 95% confidence level).

Figure 7 shows measurements of the dielectric properties ε′ and *σ* of the new broadband tissue-equivalent liquid that were made by using three different coaxial probes: the Agilent 85070E high-temperature probe (3.5 mm aperture), the Keysight 85070E Performance probe (1.6 mm aperture) and a larger probe (7 mm aperture) that was manufactured by NPL [20]. In each case, the measurement temperature was controlled at 22 ± 0.5 °C. The measurements are consistent to within the evaluations of uncertainty at coverage factor *k* = 2 (plotted as uncertainty bars). The agreement between measurements is particularly good at frequencies between 600 MHz and 4 GHz. The maximum deviation between measurements is observed at 6 GHz and is about 2.3% for ε′ and 10.9% for *σ*.

### 3.2. Evaluation of the Substitution Method of Calibration

A set of coefficients a, b and c that characterise the differences between Probe A and Probe B were obtained by the process described in Section 2.1. An alternative set of coefficients was obtained via the method described in Section 2.2 from measurements on Probe B only, following calibration at the end of the VNA cable by using GPC-7 coaxial standards (Figure 4) from a Hewlett-Packard 7-mm calibration kit model 85050A. Open-circuit, short-circuit, low-band load (<2 GHz) and a sliding load (≥2 GHz) standards were used. An Agilent 8753ES VNA was used for these measurements.

**Figure 8:**
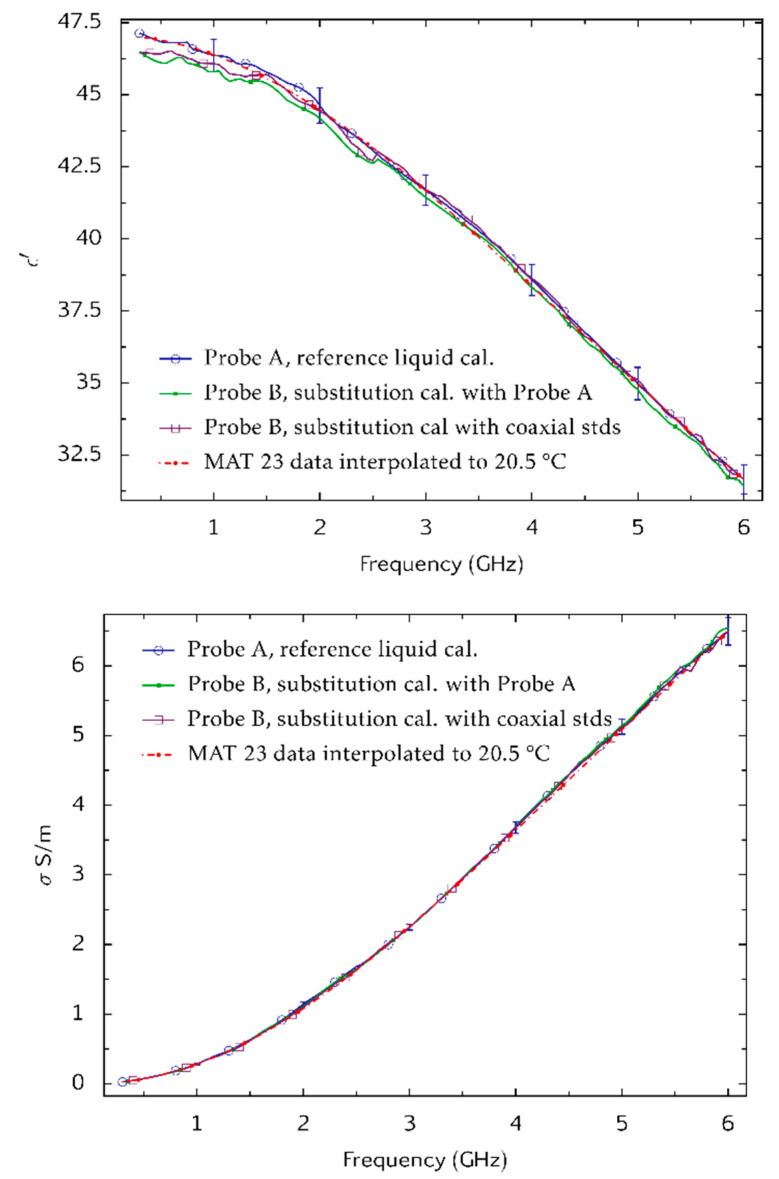
Measurements of the dielectric properties ε′ (**top**) and *σ* (**bottom**) of dimethyl sulphoxide made with Probe A (reference liquid calibration) and Probe B (substitution calibration methods). These measurements were made at NPL by using a Rohde and Schwarz model ZVB20 VNA. Reference data [21] is also shown. The measurement temperature was 20.5 °C. Combined Type A and Type B uncertainties are shown for a coverage factor of *k* = 2 (equivalent to 95% confidence level). These were evaluated by Monte-Carlo modelling [20].

**Figure 9:**
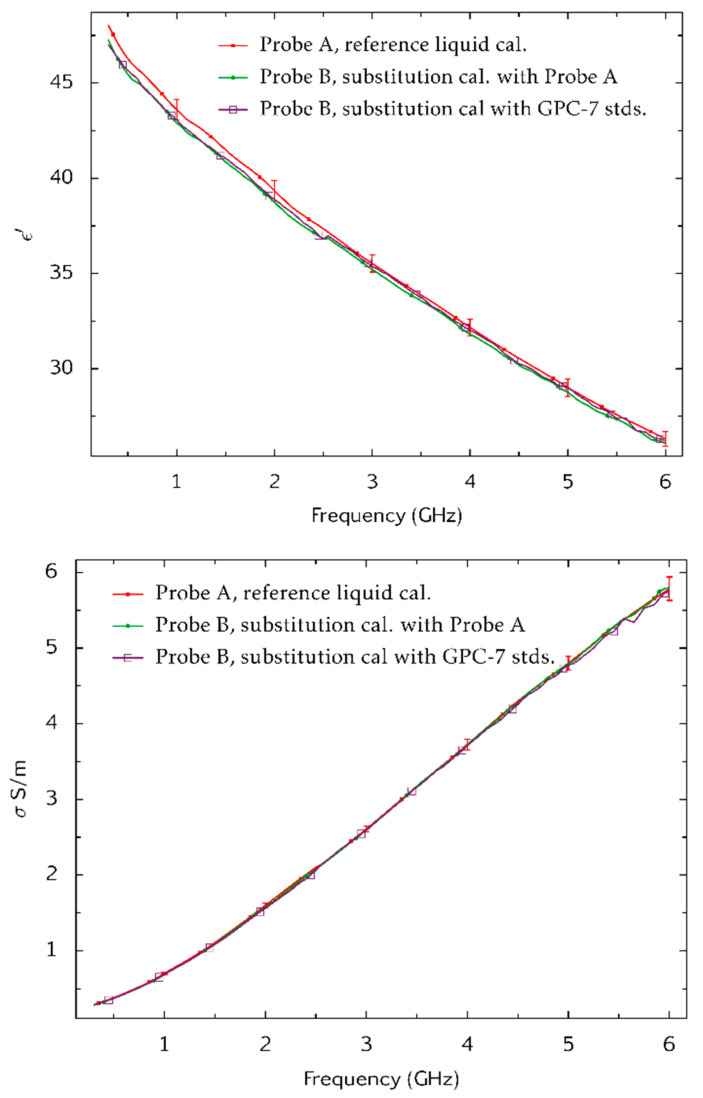
Measurements of the dielectric properties ε′ (**top**) and *σ* (**bottom**) of a tween-based tissue equivalent liquid made with Probe A (reference liquid calibration) and Probe B (substitution calibration methods). These measurements were made at NPL by using a Rohde and Schwarz model ZVB20 VNA. Combined Type A and Type B uncertainties are shown for a coverage factor of *k* = 2 (equivalent to 95% confidence level). These were evaluated by Monte-Carlo modelling [20].

A second VNA (Rohde and Schwarz ZVB20) was used for tests on the substitute-calibration process (using a different VNA provides a more thorough test for systematic errors). A high-purity reference liquid (dimethyl sulphoxide) [21] and a tween-based tissue equivalent liquid [16] were measured. Probe A was calibrated by the reference liquid method (using methanol ≥2.5 GHz and ethanol <2.5 GHz). Probe B was calibrated by substitution by (i) using the reference liquid calibration of Probe A, and (ii) using a calibration at the end of the VNA cable that was obtained with the GPC-7 coaxial standards. Measurements of ε′ and σ for each calibration are plotted in Figure 8 and Figure 9. The three measurements on each sample were made at the same temperature. Measurement uncertainties associated with the Probe A measurements were evaluated by Monte-Carlo modelling [20].

The measurements on dimethyl sulphoxide were found to be in good agreement with the reference data [21] for the three calibration methods. Measurements on the tween-based tissue-equivalent liquid made with Probe B by using the two substitute calibrations were found to agree well with the measurement made by using Probe A. It is expected that the uncertainty of measurements made with Probe B (for the substitution method of calibration using Probe A) will be increased by approximately √2 (as independent calibrations on two probes are used). This would appear to be consistent with this evaluation of uncertainty.

**Figure 10:**
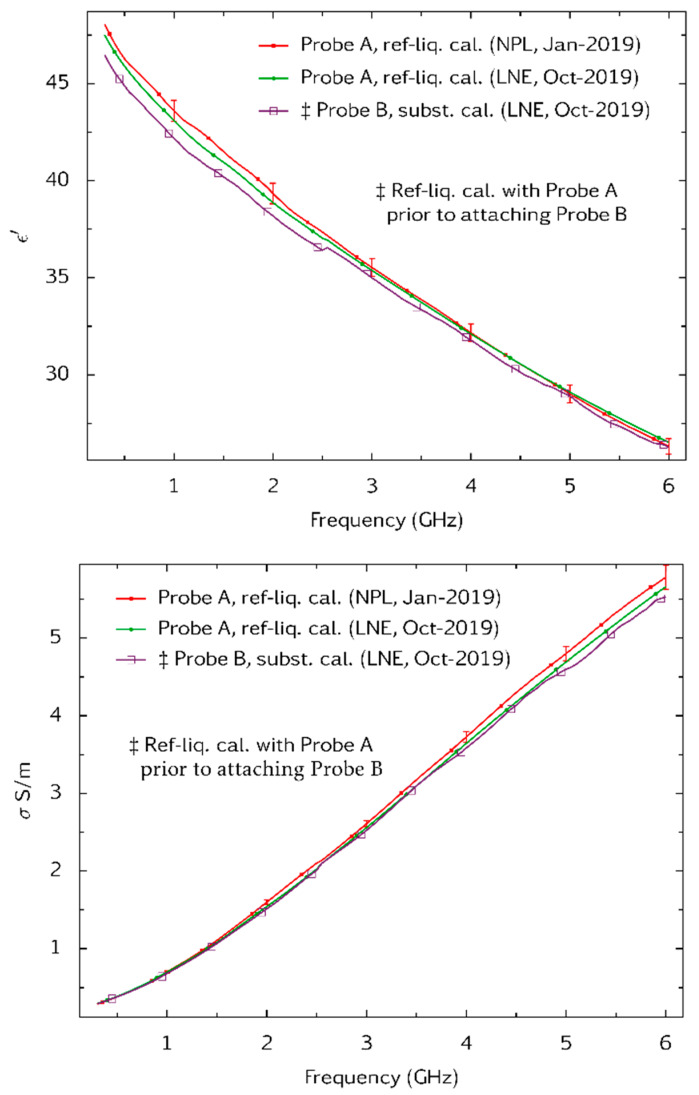
Measurements of the dielectric properties ε′ (**top**) and *σ* (**bottom**) on the same sample of a tween-based tissue equivalent liquid. Measurements with Probe A were made at NPL and, after a 9-month interval, at Laboratoire National de Métrologie et d’Essais (LNE). Probe A was calibrated at both laboratories by the reference liquid method. LNE also measured the specimen with Probe B. Calibration of Probe B was by the substitution method using the calibration of Probe A and the coefficients a, b and c in Equation (2) that were determined at NPL 9 months earlier. NPL and LNE used different vector network analyser models (a Rohde and Schwarz model ZVB20 VNA and an Agilent N5227A PNA respectively). Combined Type A and Type B uncertainties associated with the measurements obtained with Probe A are shown at a coverage factor of *k* = 2 (equivalent to 95% confidence level). These were evaluated by Monte-Carlo modelling [20].

Some 9 months after the above tests were completed at NPL, the open-ended coaxial probes, a sample of the tween-based tissue-equivalent liquid, and a file of a, b and c coefficients were shipped to the Laboratoire National de Métrologie et d’Essais (LNE) for independent tests. These used a third type of VNA (Agilent PNA N5227A). Probe A was calibrated by the reference liquid method and used to measure the same sample of tween-based tissue-equivalent liquid. The tissue-equivalent liquid was then re-measured with Probe B after applying the substitution method of calibration. The results, Figure 10, show good agreement between the techniques, and show that the tween sample has not significantly changed its properties in 9 months. Deviations between the measurements are small compared to the 10% tolerance that is specified for tissue-equivalent liquids used for SAR measurement. The viability of the substitution method for calibration is thus demonstrated.

## 4. Discussion

New vector-based SAR measurement systems require the use of a broadband tissue-equivalent liquid in a sealed tank and instrumentation for monitoring its permittivity and conductivity at microwave frequencies. For such systems to be viable, it needs to be shown that (i) the phantom liquid is stable, (ii) the liquid meets the broadband specification and (iii) the method of measurement is accurate over a sustained period of time. All three of these objectives were achieved.

As part of this work, a method of calibrating an open-ended coaxial probe embedded in a sealed tank (giving no access to the flange of the probe) has been developed. This uses a substitution method of calibration that requires the use of either GPC-7 coaxial standards, or another coaxial probe that is calibrated by the reference liquid method. An additional calibration procedure is required before the probe is embedded in the tank, but this need only be performed once if the probes remain stable. Probes manufactured for this work were found to give accurate readings of permittivity and conductivity over a 9-month period. The method can be used for industrial process monitoring where measurement of the dielectric properties of liquids in a tank or pipe are needed.

## Figures and Tables

**Table 1:** Evaluations of the Type A uncertainty associated with dielectric measurements on the broadband phantom liquid made by using the Agilent 85070E 3.5 mm coaxial probe, presented at coverage factor *k* = 2 (equivalent to 95% confidence level).

Frequency Ranges	Relative Permittivity Uncertainty (ε′)	Conductivity (σ) Uncertainty (S/m)
600 MHz to 1 GHz	1.92%	3.72%
1 GHz to 3 GHz	2.43%	4.69%
3 GHz to 5 GHz	2.81%	7.42%
5 GHz to 6 GHz	2.35%	7.92%
